# Relationships between locus of control and adherence to diabetes regimen in a sample of Iranians

**DOI:** 10.4103/0973-3930.60009

**Published:** 2010

**Authors:** Mohammad Ali Morowatisharifabad, Seid Saeed Mazloomy Mahmoodabad, Mohammad Hossein Baghianimoghadam, Nooshin Rouhani Tonekaboni

**Affiliations:** Department of Control of Disease, School of Health, Yazd Shahid Sadooghi University of Medical Sciences, Islamic Azad University; 1 Department of Health Services, Islamic Azad University; 2 Department of Nursing, Islamic Azad University

**Keywords:** Adherence to diabetes regimen, locus of control, Iran

## Abstract

**Background:**

Adequate self-care in diabetes improves quality of life and decreases the number of inpatient cases. The health locus of control theory is used to assess adherence to diabetes regimen in some studies in developed countries. The primary purpose of this cross-sectional study is to determine the status of diabetes locus of control in a sample of diabetic patients in Iran as a developing country. We investigated selected factors contributing to locus of control and adherence to diabetes regimen.

**Materials and Methods:**

This cross-sectional study was carried out on 120 patients referred to Yazd Diabetes Research Center. The Iranian versions of Diabetes Locus of Control scale and Diabetes Self-care Activities scale were used for data collection.

**Results:**

Men revealed more internal locus of control and women revealed more chance locus of control. The attributions of external locus of control increased by age, while the internal locus of control increased by education level and chance locus of control decreased by education level. A positive association between internal locus of control and adherence to diabetes regimen was found and there was a negative association between chance locus of control and adherence to diabetes regimen.

**Conclusion:**

Findings suggest that interventions aimed at improving internal locus of control may improve adherence to diabetes regimen but different diabetic patients have different attribution styles and interventional programs to enhance diabetes self-care will be more successful if patient's locus of control is addressed.

## Introduction

The diabetes regimen is extremely complex.[1] It is generally accepted that a patient with a more complex regimen is less likely to be adherent than a patient with a less demanding regimen.[2] It is crucial that individuals with diabetes follow a strict treatment regimen in order to maintain control over their blood sugar. This regimen includes maintaining a proper diet, engaging in regular physical activity or exercise, blood glucose monitoring, and taking any prescribed medications. Following this regimen may result in better self-management of diabetes and may lower the risk for experiencing chronic complications. The diabetes regimen consists of various behavior and lifestyle changes for incorporating diet, exercise, blood glucose monitoring and medication usage in one's daily life.[3] The high incidence of complications in individuals with diabetes indicates that adherence to the diabetes regimen is an eminent problem. Furthermore, it has been estimated that about 20% of individuals with type 2 diabetes do not monitor their blood glucose,[4] and only about 30% of individuals adhere to their exercise program.[5]

Adherence has been defined as the degree to which a patient's voluntary behavior corresponds with the clinical recommendations of health care providers.[6] Adherence suggests that patients are self-sufficient individuals who assume an active and voluntary role in defining and achieving goals for their medical treatment.[2] Kavanagh[7] suggests that in order to increase adherence to the diabetes regimen, it is important to determine what predicts an individual's ability to maintain the treatment objectives after the initial diabetes education program. Determining reliable predictors of adherence may allow for a better understanding of how to improve adherence to this regimen.[3]

Various psychosocial variables have been previously examined to determine their influence on adherence to the diabetes regimen. Predictors such as personality, family behaviors, health beliefs, demographic characteristics[8] and beliefs about personal control and social support[9] have been investigated. In addition, the Transtheoretical Model, the Theory of Reasoned Action, and the Health Belief Model, have been employed in the past as theoretical frameworks for investigating health behavior change and adherence to the diabetes regimen.[10–13] Although results from these studies vary, they do merit the investigation of additional psychosocial constructs as possible predictors of adherence to the diabetes regimen.

The locus of control theory was developed by Rotter.[14] The concept “locus of control” refers to the belief individuals have in the amount of control they have over their lives. Control orientation, which describes to what extent one's actions are instrumental to goal attainment, was first measured in Rotter's internal–external [I–E] scale. Individuals with high internal scores were reported to be more likely than externals to exert efforts to control their environment and to take responsibility for their actions. An external locus of control orientation indicates that goal attainment is attributed to external factors outside the control of the individual. The external orientation has been divided into “powerful others” and “chance”.[15]

Rotter's[16] Social Learning Theory employs locus of control as a generalized expectancy. Generalized expectancies are applicable in situations in which an individual has not had enough experience in a particular behavior or task to develop specific expectancies.[14,17] Therefore, locus of control is applicable in more general or novel situations.

Health locus of control is defined as a generalized expectation about whether one's health is controlled by one's own behavior or forces external to oneself.[18] Health locus of control is comprised of two components, internal locus of control and external locus of control. An individual with an internal locus of control believes that outcomes are a direct result of his or her own behavior. An individual with an external locus of control believes that outcomes are a result of either chance or powerful other people, such as physicians.[19]

The health locus of control theory is used to assess adherence to diabetes regimen in some studies. According to Rodin,[20] an individual with high perceived control may have better health because he or she is more likely to take health-enhancing actions. That is, perceptions of control influence whether an individual tries to prevent and remedy their own health problems. Therefore, a person perceives more control over their health when the locus of control is internal than when the locus of control is external.[18] This would suggest that enhancement of an individual's perceived control over his or her health may lead to improved personal health. In particular, individuals with diabetes may adhere more closely to their regimen if they experience an increase in perceived or internal locus of control. Indeed, research that has examined the relationship between perceptions of control and adherence to the diabetes regimen has found supporting evidence for the relationship between these two variables.

In a study, Macrodimitris *et al*.[21] examined the relationship between perceived control and HbA1c levels in 115 individuals with type 2 diabetes. Results indicated that perceived control was negatively related to HbA1c levels. Therefore, high-perceived control has a beneficial affect on individuals with type 2 diabetes, as demonstrated by lower HbA1c levels. It was concluded that one's perception of control over his or her condition is a good indicator of whether or not that individual will actually exhibit control over his or her condition. A study by Surgenor *et al*.[22] investigated the relationship between sense of control and metabolic control in 96 females with diabetes. Results were similar to those from Macrodimitris'.

Although the above studies have shown an association between LOC and adherence to diabetes regimen, some others have found no link between them.[12,23–25] Moreover, these studies were conducted in western countries and there is no support for these findings from developing countries. Therefore, it was thought to be beneficial to examine the relationship between locus of control and adherence to diabetes regimen in a developing country. Then, the primary purpose of this study was to determine if perceptions of control for overall diabetes management were related to adherence to the diabetes regimen in a sample of diabetic patients in Iran.

## Materials and Methods

### Procedures

This was a cross-sectional study. The non-probability sample consisted of 120 diabetic patients at Yazd Diabetes Research Center (DRC) in central Iran.

### Inclusion criteria:

1. A recognized diabetic patient for at least three months,
2. Patient having a medical file at Yazd DRC,
3. Able to speak Farsi and
4. No severe mental disorders and Alzheimer's disease.

A power analysis showed that 100 was the sufficient sample size to achieve a power of 0.80.[26] To ensure sampling adequacy, 120 diabetic patients were recruited. Participation was voluntary, and the study took place from July to August 2006. The permission to conduct the study was obtained from the Committee for the Protection of Human Subjects at the Shahid Sadooghi University of Medical Sciences and Health Services in Yazd. The investigator attended the Yazd DRC to oversee data collection. Data were collected using a one-time face-to-face private interview with all of the participants and were analyzed using the Statistical Package for the Social Sciences (SPSS). The statistical analyses of data included bivariate correlations, *t-test*, ANOVA with Tukey's HSD post hoc and linear regression with Enter method.

### Instrumentation

The survey instruments consisted of a demographic data form, Diabetes Locus of Control scale[27] and diabetes self-care activities scale.[28] The demographic data form was used to gather participants' age, gender, job, education level, marital status, type of diabetes and duration of diabetes.

The Diabetes Locus of Control scale[27] was developed for use on participants aged between 18 and 80 years. The scale consists of 18 items: Six items measuring internal locus of control, six items measuring powerful others locus of control, and six items measuring chance locus of control. A 6-point Likert-type scale is used in which 0 indicates ‘strongly disagree with the statement’ and 5 indicates ‘strongly agree with the statement’ resulting in a theoretical range of 0 to 30 for each subscale. The Diabetes Locus of Control scale has been used with success on young people as part of the Diabetes Control and Complications Trial,[29] and was considered appropriate for use with the participants of this study. The scale was translated into Farsi by the investigator. A back-translation technique[30] was used to achieve a Farsi translation which preserved the denotation and connotation of each of the instrument items. The back-translated copy was compared to the original English by investigator to recognize incongruities. The Farsi translation was then adjusted with corrective re- translation, as necessary. The Farsi version of the scale was submitted to a panel of experts to evaluate its content validity. The panel consisted of five health educators with doctoral education and extensive academic expertise in health-related areas of study. All five approved the content validity of the instrument.

The instrument was then pilot-tested with a group of diabetic patients (n = 30) to collect data to examine the internal consistency of the scales. Specifically, Cronbach's Coefficient Alpha was computed for each of the scales. The reliability coefficients were 0.76, 0.67 and 0.79 for the internal locus of control, powerful others locus of control and chance locus of control scales, respectively. For the actual study, these indices were 0.80, 0.65 and 0.82, respectively.

Adherence to regimen was measured, using the Diabetes Self-care Activities scale.[28] This measure allows participants to report how well they are adhering to their specific regimen. This is a 12-item self-report recall measure of adherence over the past seven days to five aspects of the diabetes self-care regimen, namely, 1) healthy diet, 2) insulin injecting, 3) blood glucose testing, 4) exercise and foot care and 5) smoking behavior.

The participants circle how many of the past seven days they have adhered to their prescribed regimen on each of the above behaviors. Mean scores are collected for each self-care behavior and a total adherence score can be obtained by summing the mean subscale scores.[31] In this study, the smoking behavior scale was omitted in computing adherence to regimen score because only 3% of participants reported a history of smoking behavior. The total possible scores ranged from 0 to 77, higher the score, the greater the adherent. Procedures for validation of the scale were the same as Diabetes Locus of Control scale and were carried out at the same time. The scale yielded a Cronbach's alpha of 0.66 in the pilot study and 0.68 in the actual study.

## Results

The 120 study participants ranged in age from 17 to 73 (mean = 53.28, SD = 10). Majority (60.8%) were female, all were married. Their education levels were 33.3% illiterate, 17.5% had reading and writing ability, 27.5% had primary school education and 21.7% had higher than primary school education. Most of the participants were housewives (60.8%). The overwhelming majority of the participants (82.5%) had type 2 diabetic and the rest had type 1 diabetic. The duration of diabetes ranged from three months to 30 years, with 9.8 as the average age and 6.8 as the standard deviation.

The mean score for Adherence to regimen was 48.4 (SD = 10.0). With regard to the LOC dimensions, Internal LOC solicited the highest score with an average of 26.6 (SD = 3.2), followed by Powerful others LOC and chance LOC with the means of 23.2 (SD = 2.6) and 9.4 (SD = 6.6) respectively.

A series of *t-test* for independent samples showed that 1) men significantly outscored women on the basis of internal locus of control, 2) women significantly outscored men on the basis of chance locus of control and 3) gender differences on the basis of powerful other locus of control were not statistically significant. None of the type of diabetes differences on the basis of locus of control scales scores was statistically significant. A series of one-way analysis of variance showed that type of job differences on the basis of internal and chance locus of control scales scores were statistically significant, and in both cases, Tukey's HSD post hoc procedure showed that the differences between housewives and self-employed were statistically significant. Results are summarized in Table 1.

**Table 1:** Means and standard deviations for locus of control subscales scores by gender, type of diabetes and participant's job

Variables	Values	Internal LOC	*P* value	Powerful others LOC	*P* value	Chance LOC	*P* value
Gender	Female	26.05 ± 3.13	0.001	23.36 ± 2.67	N.S	11.08 ± 7.49	0.004
	Male	27.57 ± 3.13		22.97 ± 2.66		6.78 ± 4.01	
Type of diabetes	Type 1	26.38 ± 2.99	N.S	23.47 ± 2.35	N.S	10.33 ± 7.19	N.S
	Type 2	26.70 ± 3.26		23.16 ± 2.37		9.24 ± 6.57	
Job	Employed	27.27 ± 4.02	0.008	23.18 ± 2.68	N.S	7.09 ± 3.40	0.000
	Self employed	27.80 ± 2.06		22.88 ± 2.72		5.60 ± 3.04	
	Housewives	26.06 ± 3.15		23.34 ± 2.67		11.45 ± 7.49	

Pearson Product Moment Correlation Coefficient (Pearson r) was used to describe the magnitude and direction of the bivariate associations between Adherence to regimen scores and locus of control subscales scores. Results are summarized in Table 2.

**Table 2:** Correlations among variables

Constructs	1	2	3	4	5	6
Adherence to regimen	1					
Internal LOC	0.278**	1				
Powerful others LOC	0.156	0.218*	1			
Chance LOC	−0.191*	−0.181*	0.1	1		
Age	0.178	0.124	−0.181*	−0.230*	1	
Duration of diabetes	0.177	0.098	−0.048	−0.072	0.359**	1	

* *P* < .05;

** *P* < .01

Spearman rank order correlation coefficient showed a statistically significant positive correlation between internal locus of control and level of education (r = 0.216, *P* < .05) and a statistically significant negative correlation between chance locus of control and level of education (*r* = −0.192, *P* < .05)

Correlations among variables in men and women were different. Although there were no statistically significant correlations between adherence to regimen and locus of control subscales among men, the internal and powerful others locus of control scale scores were positively correlated with adherence to regimen among women (r = 0.451 and r = 0.251, respectively). Additionally, when type 1 diabetic patients were excluded, statistically significant correlations between adherence to regimen and internal and chance locus of control were observed (r = 0.295 and r = −0.228, respectively).

Regression analysis was performed to explain variation on adherence to diabetes regimen on the basis of internal and chance locus of control. As powerful others locus of control was not significantly associated with adherence to diabetes regimen, it was not included in regression analysis. The two variables together accounted for 9.8% of the variation. However, internal locus of control was the only statistically significant predictor of adherence to diabetes regimen. Results are summarized in Table 3.

**Table 3:** Results of the regression analysis of internal and chance locus of control as predictor of adherence to diabetes regimen

Predictor	F	Beta	R^2^	*P* value
Internal LOC	6.351	0.252	0.098	0.006
Chance LOC		−.145		0.107

## Discussion

This study evaluated the status of diabetes locus of control among diabetic patients and its related factors and also quality of its association with adherence to diabetes regimen in a cross-sectional design in Iran as a developing country.

Subjects displayed internal locus of control, followed by powerful others and chance locus of control. These finding indicate that the participants considered themselves to be the greatest influence on their adherence to diabetes regimen. The findings, are similar to some studies in developed countries.[13,32–33]

The study's male participants demonstrated more internal locus of control, whereas the females displayed evidence of chance locus of control. Aalto and Uutela[12] did not find any difference in locus of control by gender but in a study by Buckelew *et al*.[34] the younger male patients reported a stronger internal attribution style and older male patients relied more heavily on both chance and powerful other factors. Since majority of the Iranian women are housewives, it was expected that housewives reported more chance locus of control and less internal locus of control. Moreover, the attributions of external locus of control are increased as age increases. Additionally, internal locus of control is increased as education level increases, while chance locus of control is decreased as education level increases. On the other hand, a positive association between internal locus of control and adherence to diabetes regimen was found and there was a negative association between chance locus of control and adherence to diabetes regimen. This is similar to the findings of previous studies[20–22,35] in developed countries.

Despite the cultural and economic differences between developed and developing countries, it seems that there is not major difference regarding the status of LOC as well as its role in determining adherence to diabetes regimen. Low level of prediction of variances in adherence to diabetes regimen by LOC attribution style indicated that the phenomenon of adherence is extremely complex and other psychosocial variables should be examined together with LOC to compare their influence on adherence to the diabetes regimen.

Due to correlational nature of the study, the reader is cautioned that no causal inferences are drawn. Moreover, the study population had low education level and most of them were housewives. Therefore, the results may not necessarily apply to all populations in developing countries. We recommend further studies in developing countries

These findings suggest that interventions aimed at improving internal locus of control may improve adherence to diabetes regimen but different diabetic patients have different attribution style. Counselors and educators should attend to the locus of control in their interventional courses and programs. The following activities will enhance internal locus of control attribution and could be used in interventional programs.

1. Providing situations which may encourage diabetic patients for adherence to regimen.
2. Providing positive feedback to patients for their small successes, as any feeling of success may make them feel that they are in control of their illness.
